# The Role of Immune Modulatory Cytokines in the Tumor Microenvironments of Head and Neck Squamous Cell Carcinomas

**DOI:** 10.3390/cancers14122884

**Published:** 2022-06-11

**Authors:** Nobuo Kondoh, Masako Mizuno-Kamiya

**Affiliations:** 1 Department of Oral Biochemistry, Asahi University School of Dentistry, Mizuho 501-0296, Gifu, Japan; 2 Chemistry Laboratory, Department of Business Administration, Asahi University School of Business Administration, Mizuho 501-0296, Gifu, Japan; mkamiya@alice.asahi-u.ac.jp

**Keywords:** cytokine, head and neck squamous cell carcinoma (HNSCC), immune modulation, tumor microenvironment (TME)

## Abstract

**Simple Summary:**

Malignant phenotypes of head and neck squamous cell carcinomas (HNSCCs) are regulated by the pro- and anti-tumoral activities of immune modulatory cytokines associated with tumor microenvironments (TMEs). We first present the immune modulatory effects of pro-inflammatory cytokines, pro- and anti- (pro-/anti-) inflammatory cytokines, and anti-inflammatory cytokines upon HNSCC phenotypes. We then report our evaluation of the functions of cytokines and chemokines that mediate the crosstalk between tumors and stromal cells, including cancer-associated fibroblasts (CAFs), myeloid-derived suppressor cells (MDSCs), plasmacytoid dendritic cells (pDCs), and tumor-associated macrophages (TAMs). In HNSCCs, the status of lymph node metastasis is an important hallmark of a worse prognosis. Several chemokines mediate lymph node metastases in HNSCC patients. There are therapeutic approaches, using antitumoral cytokines or immunotherapies, that target cytokines, chemokines, or signal molecules essential for the immune evasion of HNSCCs. Finally, modulation by human papilloma virus (HPV) infection in HNSCC phenotypes and the prognostic significance of serum cytokine levels in HNSCC patients are discussed.

**Abstract:**

HNSCCs are the major progressive malignancy of the upper digestive and respiratory organs. Malignant phenotypes of HNSCCs are regulated by the pro- and anti-tumoral activities of the immune modulatory cytokines associated with TMEs, i.e., a representative pro-inflammatory cytokine, interferon (IFN)-γ, plays a role as an anti-tumor regulator against HNSCCs; however, IFN-γ also drives programmed death-ligand (PD-L) 1 expression to promote cancer stem cells. Interleukin (IL)-2 promotes the cytotoxic activity of T cells and natural killer cells; however, endogenous IL-2 can promote regulatory T cells (Tregs), resulting in the protection of HNSCCs. In this report, we first classified and mentioned the immune modulatory aspects of pro-inflammatory cytokines, pro-/anti-inflammatory cytokines, and anti-inflammatory cytokines upon HNSCC phenotypes. In the TME of HNSCCs, pro-tumoral immune modulation is mediated by stromal cells, including CAFs, MDSCs, pDCs, and TAMs. Therefore, we evaluated the functions of cytokines and chemokines that mediate the crosstalk between tumor cells and stromal cells. In HNSCCs, the status of lymph node metastasis is an important hallmark of a worse prognosis. We therefore evaluated the possibility of chemokines mediating lymph node metastases in HNSCC patients. We also mention therapeutic approaches using anti-tumoral cytokines or immunotherapies that target cytokines, chemokines, or signal molecules essential for the immune evasion of HNSCCs. We finally discuss modulation by HPV infection upon HNSCC phenotypes, as well as the prognostic significance of serum cytokine levels in HNSCC patients.

## 1. Introduction

In Japan, 23,000 patients have been reported to suffer from cancers of the oral cavity, pharynx, and larynx. Among them, 31% are predicted to die as a direct result of the disease every year [1]. For these patients, surgery with or without radiotherapy has been widely accepted as the standard treatment; however, recent advances, i.e., immune checkpoint inhibitors as well as molecular targeting drugs, could open opportunities for alternative therapeutic interventions, including some palliative treatments [2,3]. Among squamous cell carcinoma tissues, there are variations in the contents of the TME. For example, in esophageal squamous cell carcinomas (ESCCs), cellular components are dominated by exhausted (i.e., functionally impaired) T cells and immune suppressive cells, such as Tregs, MDSCs, and TAMs [4]. Lung SCC (LUSC) tissues are infiltrated by CD45^+^ immune cells that occupy over 50% of the tumor tissues. These CD45^+^ cells, consisting of B and T lymphocytes, are three times larger in number than those of normal tissues. The T-cell subsets in LUSCs are composed of increased levels of Tregs [5]. On the other hand, the TMEs of NHSCCs are enriched in non-T immune cells, harboring chronic inflammation induced through the expression of pro-inflammatory and pro-angiogenic cytokines, which easily recruit suppressive immune cells, including MDSCs and TAMs [6,7]. In this report, we focus primarily on the cytokines and chemokines that affect the immune modulation of HNSCCs. CD4^+^ T cells are grouped into subsets, known as Th1, Th2, Th9, Th17, and T follicular effector cells. These T-cell subsets can promote different types of cytokines. However, based on their physiological characteristic, cytokines produced by T helper cells are conventionally classified into types Th1, Th2, and Th17 [8]. As a comprehensive classification of cytokines due to their inflammatory activity, they are also classified as pro-inflammatory or anti-inflammatory: pro-inflammatory cytokines favor inflammation, while anti-inflammatory cytokines function as reciprocal regulators against their pro-inflammatory opposites [9]. HNSCC development is often correlated with immune escape that depends on Tregs [10]. Th1 cells are characterized by the production of IFN-γ, which is also classified as a pro-inflammatory cytokine, and possess pleiotropic functions. IFN-γ plays a role as an antitumor regulator against OSCCs; however, IFN-γ can also promote the immune checkpoint inhibitor PD-L1 in a dose-dependent manner [11]. IL-2 results in enhanced anti-tumoral immunity in vitro as well as in vivo by promoting several immune cells; by contrast, it can also promote growth and protect HNSCC cells from apoptosis [12]. In addition, IL-2 may protect tumor cells from immune surveillance, perhaps via the promotion of Tregs [13]. The function of IL-17 can vary depending on the cell types: a higher frequency of IL-17^+^ Th cells is correlated with an improved prognosis, while IL-17^+^ non-T cells or IL-17^+^ Tc cells with Tregs in the TME are correlated with a poor prognosis [14]. These observations revealed that some pro-inflammatory cytokines share pro- and anti-tumoral activities, perhaps depending on the TME or expressed cell types.

In this review, we discuss the functions of the major immunomodulatory cytokines associated with the TME and evaluate their pro-tumoral or anti-tumoral activity for HNSCCs. Since the topic of this Special Issue is “Inflammatory Cytokines and Chemokines in Cancer”, we tentatively classify these functions as pro-inflammatory, anti-inflammatory, and as pleiotropic pro-/anti-inflammatory groups, respectively. We also report cytokines that participate in crosstalk among tumor and stromal cells, including CAFs, MDSCs, and plasmacytoid dendritic cells.

## 2. Pro-Inflammatory Cytokines

### 2.1. IFN-γ

As a representative pro-inflammatory cytokine, IFN-γ plays the role of an anti-tumor regulator against OSCCs as a result of the induction of apoptosis mediated by endoplasmic reticulum (ER) stress, unfolded protein response (UPR) mechanisms, and the downregulation of heat shock proteins or specific protein products [15].

IFN-γ is multifunctioning, depending on the effector cells, because IFN-γ expression in tumor-infiltrating lymphocytes (TILs) was correlated with a high density of TILs in the TME and also correlated with the absence of budding activity. There was also a potential correlation with the epithelial–mesenchymal transition (EMT). Morphologic correlates of the EMT, associated with the downregulation of human leukocyte antigen (HLA) and decreased inflammation, are inversely correlated with IFN-γ [16]. An immunohistochemical analysis demonstrated that the expression of IFN-γ decreased gradually with the development of oral leukoplakia (OLK) towards the lowest expression of OSCCs [17]. We have also reported a disparity in the IFN-γ-producing capability of lipopolysaccharide (LPS)-stimulated peripheral blood cells from OSCC patients, in which the IFN-γ-producing capability was high among patients at stage I but was decreased among patients whose tumor progressions were at stages II and III. Despite this, however, the highest level of IFN-γ production was seen in stage IV patients with lymph node metastases [18].

However, IFN-γ can also promote pro-cancerous activity. IFN-γ regulates the phosphorylation of STAT1/STAT3 via protein kinase D3 (PKD3), resulting in the promotion of PD-L1 in OSCC cells [19]. IFN-γ drives PD-L1 expression in a dose-dependent manner [11], and cancer stem cell markers are associated with PD-L1 expression and immune escape in OSCCs [20].

### 2.2. IL-2

IL-2, originally discovered for its immunoenhancing role in supporting the production and survival of T cells, as well as stimulating the proliferation of activated CD4^+^ and CD8^+^ effector T cells [21], can at the same time boost natural killer cell cytotoxicity [22] and augment B-cell proliferation as well as antibody secretion [23]. Since cellular immunity is commonly suppressed in HNSCC patients, the administration of IL-2 leads to improved anti-tumoral immunity in vitro as well as in vivo, but this might cause severe side effects [24]. In order to prevent side effects of serological administration, gene therapy models using IL-2 have been reported. Concerning IL-2, there are several reports on the potential of cytokine gene therapy using autologous IL-2 cDNA in a xenograft model [25]. Effective anti-tumor responses are induced by vaccinia viruses that encode exogenous IL-2 against an orthotopic murine model of HNSCC [26,27]. Liposome-mediated IL-2 gene transfection is an effective method to stimulate an autologous immune response [28].

Nevertheless, the role of endogenous IL-2 is controversial since IL-2 promotes growth and protects HNSCC cells from apoptosis [12]. IL-2 has a dual role in promoting a decrease, and tumor growth is impaired when melanoma-bearing mice are treated with IL-2 mutein. Consequently, the hypothesis put forward is that therapies that block IL-2 signals weaken Treg cell activity and stimulate immune responses [29].

### 2.3. IL-1α

A number of studies have shown the pleiotropic roles that IL-1α plays in immune regulation in the tumor milieu surrounding OSCCs. In the TME, IL-1α secreted from OSCC cells is not only able to promote the proliferation of CAFs but can also upregulate the secretion of cytokines, including the C-C motif ligand (CCL) 7, the C-X-C motif ligand (CXCL) 1, and IL-8, that promote cancer progression in human OSCC patients [30]. IL-1α affects CAFs and macrophages to enhance immune suppression and several other malignant phenotypes. IL-1α plays various roles, and is a contributing factor in the activation of *NF kappa B* and *AP-1* mRNAs, to the expression of IL-8, and to cell survival as well as the growth of HNSCC [31]. The immunosuppressive activity of mesenchymal stromal cells is enhanced by IL-1α from OSCC cells upon their direct contact with activated T lymphocytes [11].

In addition to the production of IL-1α, the nuclear localization of IL-1α in mouse OSCC cells was also synergistically enhanced under low-serum and hypoxic conditions, which also have the potential to increase the immunosuppressive property of mesenchymal cells in the TME [32]. OSCCs with a combined EGFR-positive and high nuclear IL-1α expression profile possess perineural invasion and have been positively linked to a worse prognosis [33]. Moreover, the use of an IL-1α-blocking monoclonal antibody in clinical trials has shown its potential as a therapeutic activity for advanced cancer patients [34].

### 2.4. IL-1β

Investigations of salivary transcriptomic and proteomic biomarkers have shown that the concentration of IL-1β in combination with IL-8 was notably higher in OSCC patients compared to that in dysplasia patients [35]. As a result of its interaction with the activation of the Smad/ID1 signal pathway, IL-1β promotes the stemness, cancer development, and distant metastasis of HNSCCs [36]. IL-1β modulates Snail, which in turn causes the downregulation of E-cadherin and the upregulation of COX-2, resulting in the promotion of the EMT in HNSCCs under inflammatory conditions [37]. COX-2 and its metabolite PGE_2_ are both important factors in the regulation of matrix metalloproteinase (MMP)-2 expression and the promotion of an invasive function [38]. Of particular interest is the fact that IL-1β, CCL22, and its receptor CCR4 (secreted from CAFs) promote cell transformation and Treg infiltration and prepare a protumor environment. As a result, a blockade of the IL-1β–CCL22–CCR4 signaling axis may have high therapeutic potential in the successful treatment of oral cancer [39]. Additionally, of note is the fact that a more favorable progression-free survival has been reported in HNSCC patients treated with cetuximab, which targets EGFRs. Patients who exhibited circulating levels of IL-1α and IL-1β demonstrated a higher efficacy of cetuximab treatment compared with those with low or undetectable levels of IL-1 ligands. Therefore, the indication here is that the treatment may be dependent on IL-1 signaling, which subsequently results in the activation of the anti-tumor immunity of NK and T cells [40].

### 2.5. TNF-α

Tumor necrosis factor (TNF)-α is an inflammatory cytokine produced by macrophages/monocytes found in acute inflammatory tissues, and this molecule is responsible for a diverse range of signaling events within cells, such as necrosis or apoptosis [41]. Reports have shown that increased levels of both serum and salivary TNF-α are present in OSCC subjects compared to healthy control and pre-malignant disease groups [42]. Moreover, recent studies have revealed that TNF-α induces the EMT to promote OSCC invasion through the NF-κB pathway, which is activated by Snail when interacting with Id2 [43,44]. This activity is mediated by the regulatory pathway of the TNF-α-induced ERK1/2-dependent pathway, resulting in *miR-450a*-mediated TMEM182 activation, which could significantly increase OSCC motility [45]. The angiogenesis and metastasis of OSCC cells could be induced by the activation of TNF-α via the depletion of interferon-induced protein with tetratricopeptide repeats 2 (IFIT2) [46].

However, the pro-tumorous activity of TNF-α is controversial, since high doses of TNF-α at 100 ng/mL could, in vitro, significantly inhibit the migration of OSCC cell lines via the *miR-765*–EMP3–p66Shc axis [47].

### 2.6. IL-17

The IL-17 family of cytokines is made up of six members, which are referred to as IL-17A, IL-17B, IL-17C, IL-17D, IL-17E, and IL-17F. At the head of the family is IL-17A, the prototype that has been linked to the pathogenesis of immune-mediated inflammatory diseases and cancer [48]. The role of IL-17A in cancer is controversial: while IL-17 activates antineoplastic cytotoxic T cells in melanoma and ovarian cancer, it also induces an immunosuppressive microenvironment in mammary tumors and breast cancer tissues. In fact, the function of IL-17 may also change according to the disease phase in pancreatic cancer: tumor growth is supported by IL-17 in the initial phases, while in the advanced phases, IL-17 promotes anti-tumor immunity [44].

TGF-β and IL-6 work in unison to produce a high frequency of IL-17-secreting CD4^+^ T (Th17) and CD8^+^IL-17^+^ T (Tc17) cells [49]. Therefore, a rise in IL-17-expressing cells, including Th17 and Tc17 cells, in the peripheral blood could serve as an important predictor of a poor prognosis for HNC patients, i.e., the 5-year overall survival rate is significantly decreased [50]. Interestingly, the IL-17 protein is also prominent at tumor invasion fronts (or budding sites), where cancer stem cells are enriched in OSCC tissues [51]. It has been pointed out that the clinical relevance of IL-17 may depend on the expressed cell types: whereas Th17 cells represent a beneficial response, neutrophil-expressing IL-17 is, on the other hand, associated with a poor prognosis. Importantly, in the tumor stroma of squamous cervical cancer, IL-17 is predominantly expressed by neutrophils (66%), mast cells (23%), and innate lymphoid cells (8%), while Th17 cells are only a minor IL-17-expressing population (4%) [52]. Similarly, a high frequency of IL-17^+^ non-T cells has been associated with a poor immune response, while the improved prognosis of HPV-positive oropharyngeal squamous cell carcinoma is correlated with higher numbers of tumor-infiltrating Th17 cells and lower numbers of IL-17^+^ non-T cells [14].

On the other hand, Tc17 cells and Tregs exhibited a close association in the tissues of HNSCCs, where most Tc17 cells fail to behave as cytotoxic lymphocytes. The strong positive correlation between the frequencies of Tc17 and Tregs is also observed in HNSCC patients [49].

### 2.7. IL-8

IL-8, also known as a chemokine named CXCL8, works as an attractant of polymorphonuclear inflammatory leukocytes expressing CXCL receptors (CXCRs). This molecule exhibits protumoral activities, including angiogenesis, survival signaling for cancer stem cells (CSCs), and the attraction of myeloid cells, which locally provide growth factors and promote immunosuppression [53]. Salivary cytokine levels, including IL-8, IL-6, and TNF-α, can be potential biomarkers for oral cancers [54,55]. Notably, there were positive correlations among the numbers of CD206^+^, PAI-1^+^, and IL-8^+^ OSCC cells in tumor tissues, suggesting that IL-8 production by OSCCs contributes to the generation of CD206^+^ TAMs [56]. In vitro experiments also demonstrate that IL-8 enhances the generation of CD163^+^ M2 macrophages from monocytes, after which the cells produce immune suppressive IL-10 [57]. IL-8, secreted by OSCCs, binds to CXCR2 exclusively in bone marrow mesenchymal stem cells (BMSCs) to facilitate migration to OSCCs [58]. In the same manner, TAMs are also strongly associated with the abrogation of anti-tumor immunity, tumor invasion, distant metastasis, and poor clinical prognoses, including shortened overall survival. Furthermore, IL-8 released by tumor cells mobilizes neutrophils from the circulating blood to the TME [59]. Neutrophils in the TME exert various biological activities, including the promotion of tumor angiogenesis and tumor cell proliferation [60,61]. As a mechanism that influences the efficacy of IL-8, the downregulation of the transcription factor forkhead box P3 (FOXP3) in neutrophils can eventually stimulate OSCC progression [62].

## 3. Pro-/Anti-Inflammatory Cytokines

### 3.1. IL-6

IL-6 is a multifunctional and pleiotropic cytokine that is secreted from varied cell types, including lymphocytes, monocytes, fibrocytes, keratinocytes, endothelial cells, and adipocytes, as well as cancerous cells. The activities of IL-6 in cell signaling are mediated by *classic signaling* *and trans-signaling using soluble adapter molecules, sIL-6Rs*. The contradictory responses of IL-6 are mediated by signaling, whereby pro-inflammatory responses of IL-6 are mediated by *trans-signaling*, and the regenerative or anti-inflammatory activities of IL-6 are mediated by *classic signaling* [63]. IL-6 is associated with aggressive and invasive tumor types. There is also a significant increase in the salivary concentrations of IL-6, IL-8, and TNF-α in patients with OSCCs as compared to patients with oral potentially malignant disorders (OPMDs) without dysplasia [55]. Moreover, the salivary IL-6 concentration was found to be significantly higher in patients with OSCCs when compared to patients with pre-malignant lesions [64]. We know that HNSCCs trigger CD34^+^ myeloid progenitor cells to produce increased levels of IL-6 [65]. Similarly, it is well documented that the production of IL-6 from peripheral blood monocytes in HNSCC patients is also elevated [66].

IL-6 is a principle factor in the practical crosstalk between tumor cells and CAFs in the TME [67]. The interaction between the HIF-α/MIF and NF-κB/IL-6 axes play a critical role in the hypoxia-induced accumulation of CD11b^+^Gr-1^+^ MDSCs and promotes HNSCC malignancies [68]. Furthermore, CD163^+^ functional TAMs can be developed in tumor-bearing IL-6 transgenic mice and have produced high levels of immunosuppressive molecules, such as arginase-1 (Arg-1), IL-10, and VEGF [69]. Therefore, IL-6-targeted therapies could exert promising curative effects for patients with HNSCCs. In fact, the inhibition of IL-6Rα and the downstream signaling pathways could be a target for a therapy of OSCC cells [67].

### 3.2. TGF-β

TGF-β is a multifunctional cytokine produced by several kinds of cancer-infiltrating cells, and promotes tumor growth by inducing angiogenesis, stemness, invasion, and the epithelial–mesenchymal transition (EMT) [70]. This molecule in Th17 cells promotes inflammation and augments autoimmune conditions, whereas TGF-β signaling in NK cells could cause immune suppression [71]. TGF-β produces an immunosuppressive TME in OSCC tissues by stimulating the production of Treg cells and CAFs, which then results in the inhibition of cytotoxic T lymphocytes (CTLs) and natural killer cells. Studies have revealed that TGF-β has also been shown to suppresses the function of antigen-specific CTLs in the priming and effector phases in vitro and may contribute to reduce CD8^+^ T cell proliferation in the tumor invasive front [72]. OSCCs mobilize BMSCs via the function of CXCL8. In turn, TGF-β, secreted by BMSCs, induces the EMT of OSCCs to encourage their proliferation, migration, and infiltration [58].

OSCCs induce TAMs via the activity of CAFs. Another study showed that the gene expression levels of ARG1, IL-10, and TGFB1 mRNA expression were increased in the TAMs, which strongly suppress T-cell proliferation. In fact, the neutralization of TGF-β, IL-10, or arginase 1 significantly restores T-cell proliferation [73]. A TGF-β1-mediated EMT could be triggered by SOX9. The invading OSCC cells express a high level of SOX9 products, while at the same time they change the expression of EMT markers, thereby suggesting that SOX9 is the EMT promoter [74]. Therefore, CAFs may promote cancer migration and invasion via the function of the TGF-β/SOX9 axis.

## 4. Anti-Inflammatory Cytokines

### 4.1. IL-4

IL-4 is an anti-inflammatory and immunomodulatory cytokine that activates TAMs. Reports have demonstrated that IL-4 and IL-2 synergize to promote STAT5 phosphorylation and IL-10 production, together with the selective proliferation of IL-10-producing Tregs, leading to the arresting of T-cell activation [75]. Serum levels of IL-4, in combination with some additional components, have been reported to be significantly elevated in HNSCC patients [76,77,78]. Additional data suggest that the salivary levels of the immunosuppressive cytokines IL-4, IL-10, IL-13, and IL-1RA could also prove to be potential biomarkers for OSCCs [79].

Another study showed that HNSCC samples contained CD133^+^ cancer-stem-like side population cells. These cells exhibited an elevated expression of IL-4, which confers multidrug and apoptotic resistance to tumor cells [80]. Human Thp-1 monocytes have been shown to polarize into M2-like TAMs via PMA, IL-6, and IL-4 treatment [81]. Reports have also indicated that the accumulation of M2-polarized TAMs into OSCC stroma results in a poor clinical prognosis. Signaling pathways, such as NF-κB, and the cytokines released in the tumor microenvironment promote bidirectional crosstalk between M2 and OSCC cells [82]. These observations suggest that the autocrine secretion of IL-4 is a potential target for designing novel anticancer drugs to prevent cancer stem cells and TAMs. In fact, the CD133^+^ side population cell sensitivity to drug treatment and apoptosis increased after neutralizing IL-4 [80]. In addition, it was reported that IL-4 receptor α (IL-4Rα) is markedly found on the surface of various human solid tumors, including HNSCCs [83]. A trial on the intratumoral administration of an IL-4Rα-lytic hybrid peptide effectively blocked tumor growth in a xenograft model of human HNSCCs in vivo [83]. When polarized into M2-like TAMs, dihydroartemisinin is confirmed to successfully prevent human Thp-1 monocytes by blocking the STAT3 phosphorylation necessary for IL-4R expression [81].

### 4.2. IL-10

IL-10 is a representative anti-inflammatory and immunosuppressive cytokine that promotes the immune escape of neoplastic cells [84]. The serum level of IL-10 has been shown to be statistically higher in patients with oropharyngeal squamous cell carcinomas infected with EBV and HPV, as well as those coinfected with EBV/HPV [85]. Regarding the expression of IL-10 in tumor tissues, studies reveal that it is significantly higher in HNSCC patients [86]. HNSCC tissues are infiltrated by plasmacytoid dendritic cells (pDCs). IL-10 from HNSCCs is a mediator to suppress the anti-tumoral response of pDCs against CpG-oligonucleotides and blocks the production of IFN-α [87]. It was also found that IL-10 gradually increased with the development of oral leukoplakia (OLK) toward the lowest expression in OSCCs, and this study noted that the occurrence of regulatory Tregs and TAMs also increased in parallel with disease progression [17]. The expression of immunosuppressive IL-10 is elevated in tongue leukoplakia tissues with high CD163^+^ macrophage infiltration in the TME; the expression of immunosuppressive IL-10 expression is elevated. Such tissues also represent notably significantly higher levels of epithelial dysplasia, abnormal Ki-67 expression, and cytokeratin 13 loss [88]. In HNSCC tissues, exosomes from macrophages promote tumor cell migration. In contrast, exosomes from HNSCC cells also stimulate the expression of IL-10 in macrophages and PD-L1 in tumor cells, which eventually forms an immunosuppressive environment [89]. Forkhead box D1 (FOXD1) plays an oncogene role in a variety of tumor types. In terms of prognoses, immunosuppressive genes, such as PD-L1, IL-10, TGFB1, and TGFBR1, were positively linked to FOXD1 expression in HNSCCs with an unfavorable outcome, i.e., increased TAM infiltration [90].

## 5. Crosstalk among Cancer and Stromal Cells

### 5.1. CAFs

Cancer-associated fibroblasts (CAFs) are a cellular component of the TME, contributing to tumor growth by secreting growth factors and supporting expansion, metastasis, and survival by modifying the extracellular matrix, supporting angiogenesis, and suppressing anti-tumor immune responses. As a major cellular component of the TME, CAFs play a central role in modifying other components, including TAMs, MDSCs, Tregs, cytotoxic T lymphocytes, and dendritic cells [91]. In HNSCCs, CAFs have been reported to educate mononuclear cells into the CD163^+^-macrophage-harboring characters of TAMs. CAF-promoting immunosuppression is mediated by TAMs [73].

Our results, using a mouse OSCC model, demonstrate that the immunosuppressive function of mesenchymal stromal cells is specifically enhanced by IL-1α secreted from primary OSCC cells [15]. Immune cells play a key role in the battle against cancer. In order to carry out their anti-tumor activities, T cells need to adequately respond to tumor antigens by forming contacts through the TME. Tumor-educated CAFs produce fibroblast activation protein (FAP). FAP^+^ CAFs can inhibit T-cell infiltration by way of their ECM, and their immunosuppressive functions are mediated by activating CXCL12 [92,93]. Furthermore, there is evidence that CAFs can be a factor in promoting an immunosuppressive microenvironment by inducing TAMs [73]. In OSCC tissues, CAFs are divided into three grades based on the expression of alpha smooth muscle actin (α-SMA): the highest grade CAFs promote CD163^+^ macrophages, which are associated with a poor prognosis [94]. CAFs effectively attract monocytes via the CXCL12/CXCR4 pathway and induce their differentiation into M2 macrophages that enhance the formation of cancer stem cell (CSC)-like cells from the OSCCs, which then, in turn, enhance OSCC proliferation with less apoptotic phenotypes [95]. CAF-educated macrophage progenitor cells decrease T-cell proliferation via factors such as MCP-1/CCL2, CXCL10, IL-6, TGF-β1, IL-10, and ARG1stat, which can recruit progenitors and modify TAM formations in an immunosuppressive TME [96,97,98]. A gene expression analysis demonstrates that a key regulator of CAFs is located on the PI3K-AKT pathway to induce an α-SMA phenotype and to confer immunosuppressive characteristics. Notably, several cytokines associated with immunosuppression, including IL-6, IL-8, IL-10, and TGF-*β*, as well as some other cytokines, are under the control of PI3K-AKT signals [99]. Thus, PI3K-AKT could be a potential therapeutic target to block immunosuppressive phenotypes of CAFs.

### 5.2. MDSCs

Myeloid-derived suppressor cells (MDSCs) are heterogenic immature myeloid pro-genitors that can also differentiate into granulocytes, macrophages, and dendritic cells. MDSCs expand in chronic or tumor-associated inflammations, exerting immunosuppressive functions. The generation of MDSCs is promoted by pro-inflammatory cytokines, including VEGF, IL-1β, IL-6, IL-17, and TNF-α [100].

MDSCs contribute to the progression of the malignancy of OSCCs [101]. Among circulating cells, percentages of MDSCs were increased in oral cancer patients, and their expansion is correlated with cancer stages. In tumor tissues, however, monocytic (M)-MDSCs were widespread in the periphery, whereas the polymorphonuclear (PMN)-MDSC subset dominated the tumor body compartment. M-MDSCs secreted factors, including IL-6, IL-1β, IL-23, and PGE2, and facilitated Th17 cell differentiation [102]. Syngeneic mouse OSCC models have also demonstrated the preferential activation of PMN-MDSC in metastasized OSCC tissues [103]. The PMN-MDSCs elevated in OSCC patients can exert strong immunosuppressive effects, via p-STAT3/reactive oxygen species, as well as abrogated T cell proliferation and IFN-γ production [104].

### 5.3. pDCs

Accumulated evidence indicates that plasmacytoid dendritic cells (pDCs) can also play an important role in tumorigenesis. This type of cell consists of a unique subgroup of dendritic cells that harbor a plasma cell morphology. The pDCs originally function as contributors to effective antiviral immunity; however, the cells have also been proven to modify innate and adaptive immunity by regulating the functions of lymphocytes, myeloid DCs, and NK cells by producing pro-inflammatory cytokines, TNF-α and IL-6, and to also play a role in cancer immunity [105].

It has been reported that, in primary HNSCC tissues, tumor cells produce a high level of CXCL12/SDF-1 and CXCL14, which promote the infiltration of tumor-infiltrating lymphocytes and pDCs [106,107,108]. Depending on the tumor microenvironments, pDCs are capable of exerting either an immunogenic or tolerogenic function [109]. Studies have shown that naturally occurring immunostimulatory pDCs, characterized by expressing OX40^+^, a TNF receptor super family, are often observed in HPV-positive HNSCCs [110]. OX40^+^ pDCs were distinguished by their immunostimulatory phenotypes, including their cytolytic function and ability to synergize with conventional DCs (cDCs) in generating potent tumor-antigen-specific CD8^+^ T cell responses [110]. However, in many solid tumors, including HNSCCs, pDCs are significantly pro-tumoral, with the reduced expression of IFN-α and cell surface costimulatory molecules, including TLRs, in which case they act as mediators of immunotolerance. As a matter of fact, the tumor-induced suppression of TLR9 in pDCs has been observed in protumoral TMEs [106]. The proliferation and invasion of oral cancer is bolstered by tumor-infiltrating pDCs through the activation of the TNF-α/NF-κB/CXCR-4 pathway, which may serve as a potential immunological target for the treatment of OSCCs in the future [111]. In addition, tumor-infiltrating pDCs could stimulate the inclusion of Tregs into the tumor microenvironment, which causes the promotion of immunosuppression and subsequent tumor expansion [108]. The induction of Tregs may be imparted by tumor-derived soluble factors, such as TGF-β, and is thought to promote tumor growth [112]. CXCL12 is also reported to promote the infiltration and mobilization of FOXP3^+^ tumor-infiltrating lymphocytes to enhance protumoral immunity [113].

As a novel approach for immunological treatment, CD317 antibodies have recently been applied to deplete pDCs in an immunocompetent transgenic HNSCC. pDC depletion in a transgenic HNSCC mouse model significantly delayed tumor growth. After pDCs were depleted, T cells were markedly revitalized, and the proportions of Tregs as well as MDSCs were decreased [114].

### 5.4. TAMs

Tumor-associated macrophages (TAMs) are critical for tumor progression by way of promoting angiogenesis, immune suppression, and chemotherapy resistance, as well as by affecting CSCs. Several malignant tumors, including HNSCCs, contain macrophages as major stromal cells in their tumor microenvironments. We have already reported in a review that TAMs harbor two distinct phenotypes, i.e., a conventionally activated M1 state and immune suppressive M2 states. In higher grade HNSCC tissues, a shift of macrophage polarization from M1 to M2 occurs [15]. As we have mentioned above, TGF-β, IL-4, IL-8, and IL-10 are the key mediators of the establishment of TAMs [17,56,73,81,82,83], and CAFs play important roles in educating TAMs [73,96,97,98]. Crosstalk between TAMs and OSCCs results in the rising expression of kinesin family member 4A (Kif4A) and the high infiltration of M2 macrophages, which in turn results in the overproduction of CCL2/ MCP-1, and CCR. These Kif4A–CCL2/CCR2–macrophage axes could account for the progression of immunosuppressive OSCCs [115]. CD44 is a mediator of hyaluronic acid in the TME. TAM-induced CD44 signaling mediates stemness via the PI3K-4EBP-SOX2 pathway, which then mediates the influence on CSC function [116].

## 6. Cytokines and Chemokines Associated with Lymph Node Metastases

In HNSCC patients, the status of lymph node metastasis is an established hallmark of a worse prognosis. Some cytokines and the related signaling pathways can coordinately be associated with the lymph node metastases of HNSCCs. In laryngeal carcinomas, homeobox C6 (HOXC6) and the TGF-β signaling pathway promote EMT and lymph node metastasis [117]; the overexpression of IL-22 and IL-22R1 was correlated with metastasis to the lymph node and clinical stages [118]. In HNSCCs, the elevation of serum IL-8 is positively correlated with higher grades and lymph node metastases [119]; the overexpression of CXCL3 promotes proliferation and migratory effects via the ERK1/2 signaling cascade [120]. Additionally, the overexpression of CXCR7 increases the secretion of TGF-β1, induces the EMT through PI3K/AKT, and, eventually, promotes cell migration as well as lymph node metastasis [121]. Inflammation in TMEs induces the CXCL9/10/11 chemokines in lymphatic endothelial cells (LECs) concomitantly with the elevation of CXCR3 in OSCCs and laryngeal squamous cell carcinoma cells.

Crosstalk between the LECs and the tumor cells through the CXCR3–CXCL11 or CXCL9 axes, followed by PI3K/AKT stimulation, promotes lymphovascular invasion [122,123]. Additionally, crosstalk between HNSCC cells and LECs enhances the expression of the CXCL5 protein, which, in turn, activates CXCL5-CXCR2 signaling to promote the invasion and metastasis of HNSCC cells [124]. As we have already indicated, tumor-infiltrating pDCs were significantly increased in the TME and were associated with tumor size and lymph node metastasis via the TNF-α/NF-κB/CXCR-4 pathway in OSCCs [111].

## 7. Immunotherapy

As for the immunotherapy of HNSCCs associated with cytokines, we have already offered a number of examples, i.e., the targeting of IL-4 using an IL-4Rα-lytic hybrid peptide [83], the inhibition of IL-6Rα [67], and IL-1α blocking via a monoclonal antibody [34]. IRX-2 is a primary-cell-derived Th1 cytokine consisting of IL-2, IL-1β, IFNγ, and TNF-α, produced by stimulating peripheral blood mononuclear cells of normal donors. The IRX-2 regimen is a standalone therapy for activating the immune system [125,126]. TGF-β produces an immunosuppressive TME in OSCC tissues. Targeting TGF-β, or TGF-β and prostaglandin E2, can be an effective immune therapy for HNSCC patients [127,128]. IL-33 is defined as an “alarmin”, an endogenous factor that is expressed during tissue and cell damage. Since IL-33 promotes Treg proliferation in nonlymphoid organs, stromal IL-33 is a potential target for the immunotherapy of HNSCC patients [129]. In HNSCCs, three pathways, STAT3, PI3K/AKT/mTOR, and Wnt, are the important signaling pathways underlying the immune evasion of HNSCCs. Therefore, targeting some molecules of these signals could contribute to facilitating immune therapies [130].

## 8. Discussion

It has been reported that 70% of oropharyngeal cancers are caused by the persistent infection of HPV [131]. However, patients with HPV-positive tumors have a better prognosis [132]. Some of the infections promote a pro-inflammatory milieu of the TME. In fact, in the TME of HPV^+^ HNSCC tissues, the infiltration of B and T lymphocytes is predominant, while that of neutrophils is reduced compared to those that are HPV^−^ ones. Consequently, the expression of pro-inflammatory cytokines, including IFN-γ, IL-2, IL-12, and IL-21, is predominant in HPV^+^ HNSCC tissues, while that of anti-inflammatory cytokines, including IL-10, IL-6, and TGF-β, is increased in HPV^−^ HNSCC tissues [133]. IL-17 increases the disease severity in patients with HPV infection, resulting in a hyperinflammatory condition due to the elevation of IL-17, which promotes lesions and tumor progression [134]. By contrast, HPV infection downregulates CXCL14, leading to a possible suppression of the chemotaxis of NK, CD4^+^ T, and CD8^+^ T cells, which in turn suppresses the anti-tumor immune response in the TME [135]. Therefore, the HPV status on HNSCCs is a double-edged sword that confers pleiotropic effects upon immune regulations.

Serum levels of some cytokines could be prognostic markers. In the HNSCCs of HPV+ patients, high levels of HMGB1 and anti-inflammatory cytokines, including IL-4 and IL-10, are a sign of immune evasion, which could result in recurrence [78]. High-dose radiation therapy of HNSCCs results in the significant induction of TNF-alpha, which can be measured in serum, and the complete tumor response to radiation has been strongly correlated with the induction of TNF-alpha levels in serum [136]. In OSCC patients, serum IL-6 levels are associated with increased tumor burden and aggressiveness, meaning that serum IL-6 can be a post-treatment prognostic marker after the treatment [137]. In addition, IL-10 is a potential predictor of a poor clinical outcome for the treatment of HNSCCs from laryngeal and pharyngeal origins [138,139].

As a unique cytokine-like factor that modulates the phenotypes of OSCCs, amphiregulin (AREG) is known as one of the ligands of the epidermal growth factor receptor (EGFR). This molecule is synthesized from a transmembrane precursor via a series of proteolytic steps that lead to the production of mature forms for secretion [140]. AREG upregulation is related to drug resistance and a failure of treatment for multiple types of cancer, including OSCCs [141,142,143]. Moreover, amphiregulin also stimulates Treg and suppresses CD8+ T-cell-mediated anti-tumor responses [144]. We also know that, in HNSCCs, high EGFR expression is associated with a low survival rate [145].

## 9. Conclusions

Representative immunomodulatory cytokines, in terms of their pro-tumoral and anti-tumoral activities, as well as therapeutic trials for HNSCCs, are summarized in Table 1, Table 2 and Table 3. Cytokines that mediate cross-talk among HNSCCs and immune suppressive stromal cells are summarized in Table 4.

In this review, we demonstrated that these cytokines have pleiotropic functions in the TME. This information could deepen understanding of the recent developments in tumor immunology. Further elucidation of the regulatory mechanisms, including the tumor–stromal interactions of tumor-associated cytokines, could help to identify important therapeutic targets for the development of precision medicine against advanced HNSCCs.

## Figures and Tables

**Table 1:** Representative pro-inflammatory cytokines relevant to pro-tumoral, anti-tumoral activities and therapeutic trials for HNSCCs.

	Pro -Tumoral Activities	Anti-Tumoral Activities	Therapeutic Trials
IFN-γ	Promotion of PD-L1 (* 11, 19)	Promotion of anti-tumor immunity (15)	
		Promotion of TILs (16, 17)	
IL-2	Protection of tumor cells from apoptosis (12)	Promotion of anti-tumor immunity (26, 27)	29
	Promotion of Treg (13, 29)	Stimulation of autologous immunity (28)	
IL-1α	Stimulation of CAFs to promote OSCCs (30)		34
	Promotion of tumor growth via IL-8 (31)		
	Immune-suppression via CAF (32)		
	Risk factor for recurrence (33)		
IL-1β	Promotion of tumor stemness (36)		39, 40
	Promotion of EMT (37, 38)		
TNF-α	Elevation of histological grading (42)	Inhibition of tumor migration (47)	
	Promotion of invasion/metastasis (43, 44)		
	Promotion of angiogenesis and tumor growth (46)		
IL-17	Promotion of cancer pathogenesis (48)		
	Deletion of cytotoxic T cells (49)		
	Potentiate pro-tumoral immunity (50, 51)		
	Regulation of neutrophil and poor prognosis (52)		
IL-8	Tumor progression (53, 54, 55)		
	Promotion of TAM (56)		
	Generation of M2 macrophage (57)		
	Promotion of EMT (60)		
	Regulation of neutrophil (61)		
	Downregulation of FOXP3 in neutrophils (62)		

* Numbers correspond to references in the text.

**Table 2:** Representative pro- and anti-inflammatory cytokines relevant to pro-tumoral and therapeutic trials for HNSCCs.

	Pro -Tumoral Activities	Therapeutic Trials
IL-6	Tumor progression (* 55)	67
	Enhanced production in poor prognosis of HNSCC (64)	
	Enhanced production by CD34^+^ progenitor cells (65),	
	by PB monocytes (66), by MDSCs (68) in HSCC patients	
TGF-β	Promotion of EMT (58, 70, 74)	
	Suppression of NK cells (71)	
	Suppression of antigen-specific CTLs (72)	
	Suppression of T cell proliferation (73)	

* Numbers correspond to references in the text.

**Table 3:** Representative anti-inflammatory cytokines relevant to pro-tumoral and therapeutic trials for HNSCCs.

	Pro-Tumoral Activities	Therapeutic Trials
IL-4	Elevation in HNSCC patients (* 76, 77, 78, 79)	81, 83
	Protection of tumor cells (80)	
IL-10	Elevation in HNSCC patients (85, 86)	
	Abrogation of anti-tumoral immunity (87)	
	Enhancement of Treg and M2 macrophage (88)	
	Activation of TAM (89)	

* Numbers correspond to references in the text.

**Table 4:** Cytokines mediating cross-talks among HNSCCs and immune-suppressive stromal cells.

Cytokines	CAF	MDSC	pDC
IL-6	* 73, 98	100, 102	105
TGF-β1	73, 98		112
CXCL12/SDF-1	92, 93, 95		106, 107, 113
IL-1β	98	100, 102	
VEGF	73	100	
IL-1α	15		
IL-10	98		
FAP	92, 93		
IL-33	73		
HGF	73		
CCL7	73		
IL-17		100, 102	
IL-23		102	
TNF-α		100	105, 106, 111
CXCL10			113
CXCL14			108

* Numbers correspond to references in the text.
